# Study on Hybrid Characteristics of Medicinally Used Cultivated *Codonopsis* Species Using Ribosomal Internal Transcribed Spacer (ITS) Sequencing

**DOI:** 10.3390/molecules23071565

**Published:** 2018-06-28

**Authors:** Li-Jun Liang, Er-Huan Wang, Yi-Chen Yang, Bing-Cong Xing, Wei Ji, Feng Liu, Zong-Suo Liang

**Affiliations:** 1 Institute of Soil and Water Conservation, CAS & MWR, Yangling 712100, China; llj@zafu.edu.cn (L.-J.L.); xingbingcong@163.com (B.-C.X.); 2 College of Landscape Architecture, Zhejiang Agriculture & Forestry University, Hangzhou 311300, China; 3 University of Chinese Academy of Sciences, Beijing 100049, China; 4 Buchang Pharmaceuticals Co., Ltd., Xi’an 712000, China; jilinweh@163.com (E.-H.W.); loftyideals@163.com (Y.-C.Y.); liufeng1720@163.com (F.L.); 5 College of Horticulture, Shanxi Agricultural University, Taigu 030801, China; jiweiputao@163.com; 6 Key Laboratory of Plant Secondary Metabolism and Regulation of Zhejiang Province, College of Life Sciences, Zhejiang Sci-Tech University, Hangzhou 310018, China; 7 College of Life Sciences, Northwest A&F University, Yangling 712100, China

**Keywords:** *Codonopsis* taxa, ITS, additive nucleotide, hybrid

## Abstract

*Codonopsis* taxa, as a traditional Chinese medicinal and edible plant, has found expanding domestic and foreign applications in recent decades. However, the poor management in germplasm resources market has inevitably caused an unnecessary hybrid of the provenances. In order to clarify the hybrid characteristics of germplasm resources in the main production area, the *Codonopsis* cultivars collected from the provinces Gansu, Shannxi, Shanxi, and Hubei of China were researched, using internal transcribed spacer (ITS) sequence technology. The confirmation of additive nucleotides based on the ITS sequencing of polymerase chain reaction (PCR) mixture was optimized and used to study the hybrid of *Codonopsis* cultivars. The results showed that when the ratio of PCR mixture increased up to 15 percent, the presence of a double peak in the sequencing electrophoresis map could be confirmed, suggesting the existence of additive nucleotides. According to the method above, 46 samples of *Codonopsis* cultivars collected during 2016 and 2017 were studied and compared with the samples collected from the year 2009 to 2010. All of the samples collected during 2016 and 2017 were hybridized and no genetic pure lines were found. In addition, the sites of variable base reduced greatly, concentrating at positions 122 and/or 226. These phenomena suggested that the genetic diversity of *Codonopsis* cultivars declined and the germplasm resources gradually converged. More attention should be paid to the reasonable exploitation and genetic breeding of *Codonopsis* taxa.

## 1. Introduction

*Codonopsis* Radix, normally called ‘Dangshen’, is prescribed as the dried root of *Codonopsis pilosula* (Franch.) Nannf., *C. pilosula* Nannf. var. *modesta* (Nannf.) L. D. Shen, and *C. tangshen* Oliv. of Campanulaceae [1]. *Codonopsis* Radix is a traditional Chinese medicinal and edible plant, strengthening the immune system, replenishing qi (vital energy), improving poor gastrointestinal function, and lowering blood pressure [1]. With a history of more than 300 years, *Codonopsis* taxa is mainly distributed in four provinces of China, including Gansu, Shannxi, Shanxi, and Hubei. Since 2006, the increased demand in the domestic and international market stimulated large-scale and regional cultivation of *Codonopsis* taxa, especially in Gansu province, accounting for 90% of the total cultivation area in China [2]. Because of the lack of professional support in seed and seedling production, most farmers buy seeds and seedlings only in pursuit of high yield. *C. pilosula* in Min County and *C. pilosula* var. *modesta* in Longnan city of Gansu Province are always selected as the germplasm resources owing to the high yield*.* Such a production model probably resulted in homogenization of germplasm resources and a decrease in genetic diversity [3,4,5]. In this study, DNA molecular markers were used to analyze the germplasm resources and genetic characteristics of *Codonopsis* taxa in the main production areas, and the hybrid characteristics of cultivated species were elucidated.

A large number of molecular marker techniques, such as simple sequence repeat (SSR), single nucleotide polymorphism (SNP), and internal transcribed spacer (ITS), have been used in plant classification, identification, and analysis of genetic characteristics [6,7,8,9,10,11,12,13,14,15]. Among them, ITS sequence of nuclear ribosomal DNA (nrDNA) has been proved to be a helpful non-coding marker to infer hybridization events [5,16,17,18,19,20,21,22]. Because ITS has relatively high levels of phylogenetically informative sequence variation [23], it can provide evidence of evolution when the hybrid retains the repeat types contributed from each parental species [24,25,26]. Additive nucleotides (also called variable base) presented in ITS sequences are often used to study the origin of hybridization parents [27,28,29,30,31,32,33,34,35,36,37,38,39,40,41]. They are formed from two or more different bases that appear simultaneously at one sequence position, and each of them represents a single parent and is heterozygous at that position. However, because of the influence of background noise from electrophoresis sequencing, it is critical to determine whether variable bases are actually present. To determine the existence of variable bases, the intensity of the primary signal, secondary signal, and noise signal of variable bases shown in the electrophoretic sequencing map are calculated and analyzed statistically.

Although molecular marker techniques have been widely used in studying the diversity of medicinal plants, ITS and SSR were used to study the diversity of *Codonopsis* taxa [4,5,19]. He JY et al. [19] studied the origin of *Codonopsis* hybrids, as well as the hybrid characteristics of *Codonopsis* collected during the year 2009 and 2010, based on the ITS gene sequence. Large-scale plantation of *Codonopsis* cultivars in the main production area after the year 2010 probably accelerated the hybrid of germplasm resources. To reveal the changes in *Codonopsis* cultivars after seven years and arouse the attention on protecting the genetic diversity of *Codonopsis* taxa, this study sequenced the ITS genes of 46 samples of *Codonopsis* cultivars from the provinces Gansu, Shaanxi, Shanxi, Hubei, and Ningxia Hui Autonomous Region of China collected during the year 2016 and 2017.

## 2. Results and Discussion

### 2.1. Verification of Additive Nucleotides

To distinguish the secondary peak at the 122nd position from noise and verify the additive nucleotides, the secondary peak (*S*)-value and background noise level (*N*)-value were calculated according to Equations (1) and (2). Table 1 presents the *S*-value of the additive peak at the 122nd position and the *N*-value of the high noise near the 122nd position in the ITS gene (*n* = 5). When the percentage of polymerase chain reaction (PCR) product of CT46 (pCT46) increased from 10% to 50%, the average *S*-value rose from 11.54% to 47.72%. The *S*-value of the secondary peak at the 122nd position was positively correlated to the amount of pCT46. Variance analysis between the *S*-value and *N*-value was performed and the significance of the difference between the two values was calculated to prove the existence of the double peaks at this position. Correspondingly, the existence of the double peaks suggested the presence of additive nucleotides. The results in Table 1 indicated that when the amount of pCT46 was 10% or less, the difference between the *S*-value and *N*-value was insignificant, and the secondary peak could not be confirmed, as well as the presence of additive nucleotides. When the amount of pCT46 increased from 10% to 15%, the existence of additive nucleotides should be detected by the significant of the difference between the *S*-value and *N*-value. When the amount of pCT46 was 15% or more, the existence of additive nucleotides could be confirmed directly. This phenomenon was also detected by Kitani Y. et al. [17] and He JY. et al. [19]. Because the *S*-value of the secondary peak was similar to the amount of the PCR product mixture, it could be used to calculate the hybrid rate. 

### 2.2. ITS Sequences of Codonopsis Plant Species

**Figure 1:**
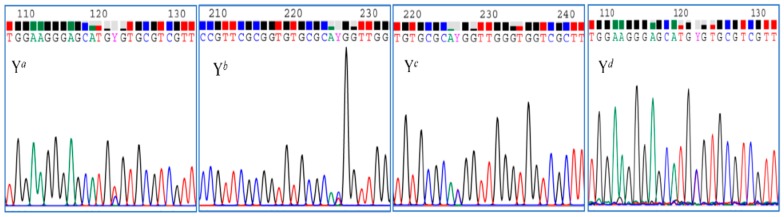
Four types of additive nucleotide Y at position 122nd or 226th. Y*^a^* indicates the additive nucleotide Y (T > C) at the 122nd position; Y*^b^* indicates the additive nucleotide Y (C > T) at the 226th position; Y*^c^* indicates the additive nucleotide Y (T > C) at the 226th position; Y*^d^* indicates the additive nucleotide Y (C > T) at the 122nd position.

The length of the ITS (ITS1-5.8S-ITS2) region was 655 bp in the selected 46 *Codonopsis* specimens, and the length of ITS1, 5.8S, and ITS2 was 257, 163, and 235 bp, respectively (Figure S1). When comparing the ITS sequences of *C. pilosula*, *C. pilosula* var. *modesta*, and *C. tangshen* in this study with those of the genus *Codonopsis* in GenBank online, high homology was found with accession numbers EF190460, EF190461, EF190462, and AB769272 (designated as P0, PM0, T0, and S0), which had been reported by Lin TC. et al. [16] and He JY. et al. [19] as the pure line. Among the four species, AB769272 is the ITS sequence of an unidentified specimen of genus *Codonopsis*. As shown in Table 2, the ITS sequences showed different variable sites in *C. pilosula*, *C. pilosula* var. *modesta*, and *C. tangshen*. One or two positions including the 122nd and/or 226th positions only appeared on ITS1 regions of *C. pilosula* and *C. pilosula* var. *modesta*, while the 135th and 500th positions presented on *C. tangshen*. The variable sites on all of these specimens were additive bases Y (Y = C and T) or R (R = A and G). The additive nucleotides Y, representing double peaks of C and T in electrophoretogram, were frequently observed at positions 122 and/or 226, especially at the former. Among them, the additive nucleotides Y (T > C) at the 122nd position accounted for a large proportion, with only four specimens, including CP26, CP34, CP37, and CP38 displaying Y (C > T) at the same position. At the 226th position, all the specimens presented additive nucleotides Y (C > T), except for the CP10 (T > C) (Figure 1).

According to the composition of the nucleotide in the ITS sequences, the 46 specimens were classified into nine ITS-type sequences. The nine ITS-type and four pure-line sequences were also used to construct the phylogenetic tree in 2.3. The tested *C. pilosula* was divided into five types, namely, P1, P2, P3, P4, and P5, the corresponding numbers of which were 33, 1, 1, 2, and 2, respectively. *C. pilosula* var. *modesta* was divided into three types according to the method above, namely, PM1, PM2, and PM3, with the numbers of 4, 1, and 1, respectively. Only one *C. tangshen* specimen (CT46) was found in this study.

As one of the main production areas of *Codonopsis* cultivars, Gansu province of China has two species, including *C. pilosula* and *C. pilosula* var. *modesta*. No genetic pure line of them collected during the year 2016 to 2017 had been found according to the ITS sequencing results. There were only five types of ITS sequences in the *C. pilosula*, and three types in the *C. pilosula* var. *modesta*. The variable base concentrated at the 122nd and/or 226th nucleotide sites, and all were located at ITS1 region. In other words, there were no variable base sites in the 5.8S and ITS2 regions. As for the ITS type of the *C. pilosula*, P1 types were dominant with a percentage near 85%, and for the *C. pilosula* var. *modesta*, PM1 approached 67%. Although the *C. pilosula* is different from *C. pilosula* var. *modesta* in morphological characteristics, the ITS sequences of P1 and PM1 were similar, which could not be used to distinguish the two species.

However, in 57 samples tested in the studies of He JY. et al. [19] during the year 2009 and 2010, genetic pure lines of *C. pilosula* and *C. pilosula* var. *modesta* were found and accounted for 13% and 47%, respectively. ITS type was 11 for *C. pilosula* and 5 for *C. pilosula* var. *modesta*. They also reported that six variable base sites presented in ITS sequences of *C. pilosula* (122nd, 130th, 226th, 441st, 489th, and 519th) and *C. pilosula* var. *modesta* (130th, 226th, 441st, 489th, 509th, and 533rd), while in our research, only the 122nd and 226th were found. Above all, the ITS type of cultivated *Codonopsis* taxa decreased, as well as the number of the variable base, which suggested a reduction of genetic diversity of the *Codonopsis* taxa. These phenomena might be related to the gradual convergence of the seedling sources and natural hybridation in the main production areas of Gansu province of China.

### 2.3. Molecular Phylogenetic Tree Based on the ITS Gene Sequences

**Figure 2:**
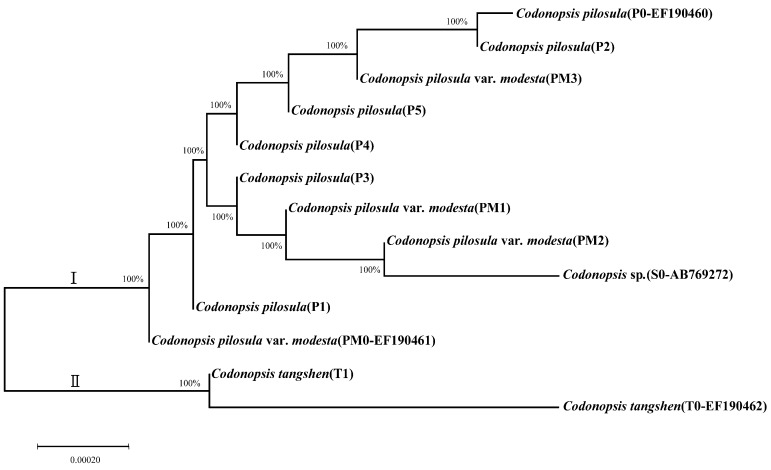
Neighbor-joining (NJ) evolutionary relationships of *Codonopsis* taxa based on internal transcribed spacer (ITS) sequences.

A molecular phylogenetic tree based on ITS was generated as shown in Figure 2. A total amount of 13 nucleotide sequences, including 9 studied specimens and 4 pure lines from GenBank, were adopted to analyze the evolutionary relationship.

The studied specimens were highly homologous to the four pure lines from GenBank (Figure 2). Two monophyletic clades (designated as Ι and II) on the phylogenetic tree were developed. Clade Ι consisted of eight cultivars and three pure lines. The eight cultivars were composed of five ITS types of *C. pilosula* and three types of *C. pilosula* var. *modesta*. Clade II consisted of one cultivar of *C. tangshen* and one pure line. The phylogenetic tree in this study showed that *C. pilosula*, *C. pilosula* var. *modesta*, and *C. tangshen* were closely related, with a relatively closer relationship between the first two. *C. tangshen* had relatively far relationship with *C. pilosula* and *C. pilosula* var. *modesta*, which agreed with the definition in the Chinese Pharmacopeia. The unidentified *Codonopsis* sp. (S0) was closely related to *C. pilosula* var. *modesta*, rather than *C. pilosula* [14]. Accordingly, we speculated that it belonged to the species *C. pilosula* var. *modesta*.

### 2.4. Hybridization Analysis Based on the ITS Gene Sequences

According to the results of the aligned ITS sequence, all of the specimens in our investigation field were heterozygous. The additive nucleotide mainly presented at positions 122 and/or 226 in the species of *C. pilosula* and *C. pilosula* var. *modesta*. No pure line was found among the 45 specimens of *Codonopsis* Taxa. Combining the relationship in the phylogenetic tree with nucleotide additivity in the ITS sequence, the parental lineages could be referred [27]. The additive nucleotides at positions 122 and 226 were probably formed from a hybrid of two pure lines (Table 2). Five types (P1–P5) of ITS sequences were found in *C. pilosula* specimens, among which P1, P3, and P4 might be produced from the heterozygosis between P0 (C C) and S0 (T T), and P5 from P0 (C C) and PM0 (T C), and it was hard to speculate the hybrid origin of P2. Three types (PM1–PM3) of ITS sequences were determined in *C. pilosula* var. *modesta*, of which PM1 probably originated from the heterozygosis of P0 (C C) and S0 (T T), PM2 from S0 (T T) and PM0 (T C), and PM3 from P0 (C C) and PM0 (T C). 

The *S*-value was used to calculate the hybrid ratio of specimens, as expatiated in 2.1. The results in Table 3 show that among the 46 specimens, up to 33 of *C. pilosula* displayed sequence type P1, and the average hybrid ratio at positions 122 and 226 were 39.01% and 33.69%, respectively. The number of specimens with sequence type P2, P3, P4, and P5 were relatively small, only 1, 1, 2, and 2, respectively. The average hybrid ratio of type P2 at the 226th position was 33.5%, and that of type P3 at positions 122 and 226 was 32.7% and 48.4%, respectively, and 36.32% and 24.52%, respectively, for type P4. The heterozygosis of type 5 only happened at the 122nd position, with an average hybrid ratio of 39.09%. Six specimens of *C. pilosula* var. *modesta* were found and classified into three types. The average hybrid ratios of PM1 at positions 122 and 226 were 34.58% and 30.85%, respectively, while that of PM2 at the 226th position was 25.53%, and of PM3 at the 122nd position was 27.35%.

Further analyses on the sequence type of *C. pilosula* and *C. pilosula* var. *modesta* manifested that types P1 (Y Y) and PM1 (Y Y) accounted for a large proportion, and the average hybrid ratios at positions 122 and 226 varied greatly, which suggested that multiple hybrids probably happened, rather than the hybrid of homozygotes P0 (C C) and S0 (T T). For example, S0 (T T) hybridized with PM0 (T C) to form PM2 (T Y), which probably further hybridized with P0 (C C) to form P1 (Y Y) or PM1 (Y Y). Additionally, PM3 (Y C) was produced from the hybrid between P0 (C C) and PM0 (T C), which could further hybridize with S0 (T T) to form P1 (Y Y) or PM1 (Y Y).

**Figure 3:**
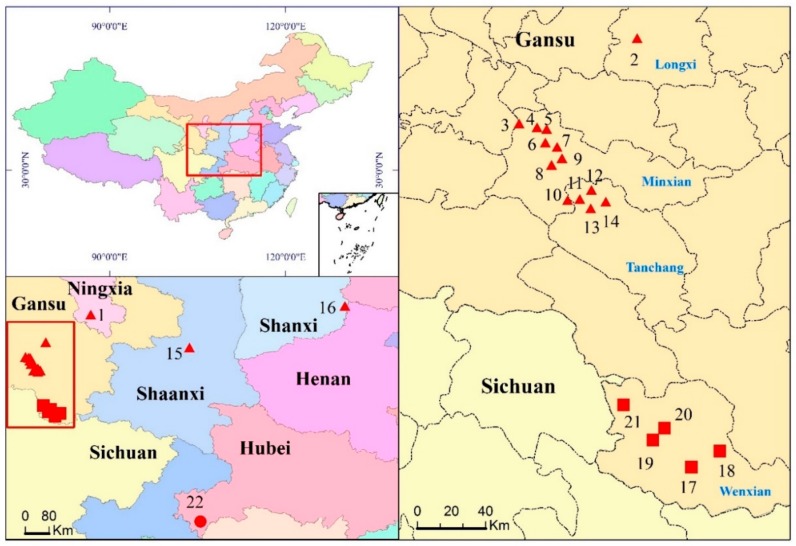
Collection sites of *Codonopsis* specimens during in 2016 and 2017. The numerals of the collection sites are indicated in Table 3. The solid triangles indicate *C. pilosula*. The solid squares indicate *C. pilosula* var. *modesta*. The solid circulars indicate *C. tangshen*.

No obvious correlation between the heterozygous rate and geographical factors, including altitude longitude and latitude (Table 4 and Figure 3), could be speculated. Therefore, this phenomenon might arise from the selection of germplasm resources, which became more monotonous in long-term artificial cultivation. It is reported that a large number of variable nucleotide sites existed in the wild *C. pilosula* [42], so much more attention should be paid to the reasonable exploitation and genetic breeding of *Codonopsis* taxa. 

## 3. Materials and Methods

### 3.1. Plant Materials

Forty-six identified *Codonopsis* specimens including 39 strains of *C. pilosula*, 6 strains of *C. pilosula* var. *modesta*, and 1 strain of *C. tangshen* were studied. They were collected from the cultivation fields of Gansu, Shanxi, Shaanxi, and Hubei province, and Ningxia Hui Autonomous Region of China during our field investigation from 2016 to 2017 (shown in Table 4). All specimens were stored in the laboratory of plant secondary metabolism and regulation of Zhejiang Province, College of Life Sciences, Zhejiang Sci-Tech University, China.

### 3.2. DNA Extraction

The genomic DNA was extracted from 50–60 mg dried roots by Plant Genprep DNA Kit (Zoman Biotech Co., Beijing, China) with minor modifications to the protocol provided by manufacturer, that is, 5–6 mg polyvinyl pyrrolidone K40 (Molecular Biology Grade, Sangon Bio-tech Co., Shanghai, China) was mixed with the dried root before grinding. Incubation time at 65 °C was extended from 30 min to 6 h [5]. The purity and quantity of extracted DNA was detected using NanoDrop-2000 (Thermo Scientific, Wilmington, NC, USA). The quality was detected by electrophoresis on 1.0% agarose gel stained with ethidium bromide. DNA samples were stored at −20 °C before using in the PCR amplification. Three replicates were prepared for DNA extraction and PCR amplification.

### 3.3. PCR Amplification

The primers used for amplification of ITS were oligonucleotide ITS5F (Forward primer 5′-GGA AGT AAA AGT CGT AAC AAG G-3′) and ITS4R (Reverse primer 5′-TCC TCC GCT TAT TGA TAT GC-3′) [3,5,43]. Amplification reaction was performed in a volume of 20 μL mixture, including 10 μL 2 × Ftaq PCR Mix (Zoman Bio-technology Co., Beijing, China), 0.25 μM of each primer, approximate 20–100 ng template DNA, and 8 μL RNase-free water. A T100 thermal cycler (Bio-Rad Laboratories, Inc., Hercules, CA, USA) was used to carry out PCR amplification under the cycling condition: initial denaturation at 95 °C for 5 min, followed by 35 cycles of denaturation at 95 °C for 30 s, annealing at 50 °C for 30 s, extension at 72 °C for 50 s, and then final extension at 72 °C for 5 min. The 5 μL of PCR product was detected by 1.0% agarose gel electrophoresis and sequencing was performed by Tsingke Bio-tech Co., Hangzhou, China. The primers in sequencing and PCR amplification were the same and two directional sequencing were conducted using ABI 3730XL sequencer (SeqGen, Inc., Torrance, CA, USA).

### 3.4. Verification on Variable Nucleotide

In ITS sequencing of *Codonopsis* specimens, double peaks were found in one position of the base, which might be produced from variable nucleotides. To confirm the presence of variable nucleotides, verification experiments were conducted referring to the previous studies with minor adjustments [17,19]. According to the ITS sequencing results of the 46 specimens, CP02 and CT46 were selected for the verification of variable nucleotide, because they had a single cytosine (C) and thymidine (T) peak at the 122th position, respectively. The ITS gene of the two specimens was amplified and the concentration of PCR products was detected. A series of PCR mixtures were prepared with the ratio of CT46 10%, 15%, 20%, 30%, 40%, and 50%, respectively, and five replicates for each mixture. All PCR samples were sequenced by Qingke Biotechnology Co., Hangzhou, China. Same primers were used for PCR amplification and sequencing as 3.3. The sequencing results were examined using the Bioedit program [44]. The relative intensities of the secondary peak (*S*) and the background noise level (*N*) at the 122nd position were manually calculated as follows.

(1) $$S-value=\frac{I_{2}}{I_{1}+I_{2}}$$

(2) $$N-value=\frac{I_{0}}{I_{0}+I_{1}}$$

where *I*_0_ is the intensity of noise peak, which is the average intensity of peaks from the 117th to 127th nucleotide sites; *I*_1_ is the intensity of the main peak at the 122nd nucleotide site; and *I*_2_ is the intensity of the secondary peak at the 122nd nucleotide site.

### 3.5. Processing on ITS Sequence 

The two directional sequences obtained by sequencing were assembled and edited using the ContigExpress program [45]. The nucleotide composition of the sequences was then analyzed and variable base sites were recorded. The presence of additive nucleotide is confirmed as follows. The possible positions of additive nucleotides in ITS sequences were inspected. If the same positions are found in the forward and reverse sequences, *S* and *N* values will be calculated based on the forward sequences using the Bioedit program [44]. If a significant difference exists in the two values, the presence of additive nucleotides can be confirmed. 

According to the reported nucleotide composition of the ITS (ITS1-5.8S-ITS2) sequence [5,16,19], it was found that the initial base of ITS contained “TCGAA” and the terminal base contained “TCCGACC”, and the 5.8S region began with “AAACGACTCT” and ended at “CGTCACGC”. Thus, the ITS gene sequence can be obtained, and the length of ITS, ITS1, 5.8S, and ITS2 can be calculated. 

The BLAST tool was used to compare and analyze the related data of the sequence and NCBI website. Based on the ITS gene sequences, the phylogenetic tree was analyzed on hierarchical clustering of the ITS alignments, and produced by MEGA 7 program [46] with neigbour-joining of the bootstrap values (1000 replicates).

## 4. Conclusions

This study optimized the method of direct sequencing of mixed PCR products to detect additive nucleotides of *C. pilosula*, *C. pilosula* var. modesta, and *C. tangshen* in the nrDNA ITS sequence. When the ratio of PCR product added up to 15%, the presence of additive nucleotides could be confirmed. Compared with *Codonopsis* specimens collected during the year 2009 and 2010, the genetic pure lines disappeared, the ITS type reduced greatly, and the variable base sites declined from six to two in the specimens collected during the year 2016 to 2017. These phenomena show that the genetic diversity of *Codonopsis* taxa in main production areas has degenerated. Therefore, measures should be taken to build germplasm resources of *Codonopsis* taxa, protecting the genetic diversity and promoting the sustainable development of the *Codonopsis* taxa industry. The results of the ITS sequencing also indicated that the ITS marker alone could not be used to identify the three *Codonopsis* taxa, other morphological classification and molecular marker technologies should be combined to identify them.

## Figures and Tables

**Table 1:** *S*-value of the additive peak at the 122nd Position and *N*-value of the high noise sites near the 122nd position in the internal transcribed spacer (ITS) gene (*n* = 5).

pCT46 (%) *^a^*	Main Signal	*S*-Value (%)	*N*-Value (%)	Significance *^b^*
10	C	11.54 ± 1.46	10.82 ± 0.98	
15	C	14.82 ± 1.16	10.64 ± 1.13	**
20	C	21.08 ± 2.23	10.66 ± 1.07	**
30	C	29.14 ± 1.65	8.92 ± 1.18	**
40	C	39.60 ± 1.87	9.02 ± 1.19	**
50	C	47.72 ± 1.37	8.84 ± 1.16	**

*^a^* indicates the percent of polymerase chain reaction (PCR) products of sample CT46 to sample CP02. *^b^* indicates the significance of difference between *S*-value and *N*-value. The *S*-value and *N*-value were equal to ‘Average ± S.D.’. The double asterisk indicates the difference analyzed between the *S*-value and *N*-value was very significant (*p* < 0.01).

**Table 2:** Types of ITS sequences of *Codonopsis* species and the assumed lineages related to hybridization.

Species	ITS Type	G + C Content (%)	Nucleotide Position				Sequence Type of Supposed Parental Lineages (Nucleotides at 122nd and 226th)	Number of Specimens	GenBank Accession No.
			122	135	226	500			
*C. pilosula*	P0	60.61	C	G	C	G			EF190460
	P1	60.31	Y *^a^*	*	Y *^b^*	*	P0 (C C) × S0 (T T)	33	
	P2	60.46	*	*	Y *^b^*	*		1	
	P3	60.31	Y *^a^*	*	Y *^c^*	*	P0 (C C) × S0 (T T)	1	
	P4	60.31	Y *^d^*	*	Y *^b^*	*	P0 (C C) × S0 (T T)	2	
	P5	60.46	Y *^d^*	*	*	*	P0 (C C) × PM0 (T C)	2	
*C. pilosula* var. *modesta*	PM0	60.46	T	*	*	*			EF190461
	PM1	60.31	Y *^a^*	*	Y *^b^*	*	P0 (C C) × S0 (T T)	4	
	PM2	60.31	T	*	Y *^b^*	*	S0 (T T) × PM0 (T C)	1	
	PM3	60.46	Y *^a^*	*	*	*	P0 (C C) × PM0 (T C)	1	
*C. tangshen*	T0	60.31	T	*	*	A			EF190462
	T1	60.15	T	R	*	R		1	
*C.*SP.	S0	60.31	T	*	T	*			AB769272

Numerals above sequence are aligned nucleotide positions. Y = C and T, R = A and G, asterisk indicates the identical nucleotide to P0. *^a^* indicates the additive nucleotide Y (T > C); *^b^* indicates the additive nucleotide Y (C > T); *^c^* indicates the additive nucleotide Y (T > C); *^d^* indicates the additive nucleotide Y (C > T).

**Table 3:** The hybrid ratio of the genus *Codonopsis* specimens based on the ITS sequences (*n* = 3).

Voucher No.	Species	Sequence Type (ITS)	Hybrid Ratio (%, 122nd)	Hybrid Ratio (%, 226th)
CP01	*C. pilosula*	P1	33.46 ± 1.29	46.89 ± 1.35
CP02	*C. pilosula*	P2		33.49 ± 1.55
CP03	*C. pilosula*	P1	37.47 ± 1.47	37.07 ± 0.50
CP04	*C. pilosula*	P1	36.14 ± 1.73	39.39 ± 1.17
CP05	*C. pilosula*	P1	48.33 ± 1.56	25.14 ± 2.52
CP06	*C. pilosula*	P1	35.23 ± 1.31	39.18 ± 1.52
CP07	*C. pilosula*	P1	33.25 ± 0.70	41.59 ± 0.45
CP08	*C. pilosula*	P1	44.77 ± 1.09	39.70 ± 1.62
CP09	*C. pilosula*	P1	31.9 ± 1.17	33.57 ± 2.13
CP10	*C. pilosula*	P3	32.72 ± 1.48	47.79 ± 1.77
CP11	*C. pilosula*	P1	40.86 ± 1.71	29.05 ± 2.04
CP12	*C. pilosula*	P1	38.34 ± 1.26	39.92 ± 2.39
CP13	*C. pilosula*	P1	35.94 ± 0.98	40.33 ± 3.32
CP14	*C. pilosula*	P1	44.52 ± 0.72	38.14 ± 1.68
CP15	*C. pilosula*	P1	35.05 ± 1.40	43.61 ± 2.30
CP16	*C. pilosula*	P1	30.65 ± 0.99	32.98 ± 2.03
CP17	*C. pilosula*	P1	32.57 ± 0.80	28.64 ± 0.82
CP18	*C. pilosula*	P1	45.63 ± 0.93	32.35 ± 2.46
CP19	*C. pilosula*	P1	39.53 ± 3.48	40.20 ± 2.11
CP20	*C. pilosula*	P1	32.24 ± 0.63	37.79 ± 1.06
CP21	*C. pilosula*	P1	40.25 ± 0.21	40.62 ± 1.27
CP22	*C. pilosula*	P1	35.93 ± 0.35	37.99 ± 1.30
CP23	*C. pilosula*	P1	39.33 ± 0.94	33.61 ± 1.03
CP24	*C. pilosula*	P1	36.48 ± 0.57	34.09 ± 1.12
CP25	*C. pilosula*	P1	39.65 ± 0.17	34.66 ± 0.83
CP26	*C. pilosula*	P4	47.64 ± 1.72	32.05 ± 2.11
CP27	*C. pilosula*	P1	34.86 ± 1.51	22.40 ± 1.51
CP28	*C. pilosula*	P1	43.02 ± 0.83	31.94 ± 0.82
CP29	*C. pilosula*	P1	40.71 ± 1.63	25.37 ± 1.88
CP30	*C. pilosula*	P1	48.02 ± 0.41	22.07 ± 1.46
CP31	*C. pilosula*	P1	43.49 ± 0.46	20.11 ± 1.74
CP32	*C. pilosula*	P1	47.36 ± 0.41	17.41 ± 1.73
CP33	*C. pilosula*	P1	28.92 ± 0.49	29.00 ± 5.90
CP34	*C. pilosula*	P5	34.88 ± 5.96	
CP35	*C. pilosula*	P1	46.22 ± 6.07	28.87 ± 1.41
CP36	*C. pilosula*	P1	43.10 ± 4.92	29.25 ± 2.70
CP37	*C. pilosula*	P5	39.67 ± 6.60	
CP38	*C. pilosula*	P4	24.14 ± 0.85	16.47 ± 0.66
CP39	*C. pilosula*	P1	34.67 ± 1.01	42.17 ± 0.71
CPM40	*C. pilosula* var. *modesta*	PM1	36.99 ± 4.01	38.67 ± 0.54
CPM41	*C. pilosula* var. *modesta*	PM1	39.29 ± 0.25	33.92 ± 1.33
CPM42	*C. pilosula* var. *modesta*	PM1	36.88 ± 0.49	21.23 ± 0.66
CPM43	*C. pilosula* var. *modesta*	PM1	22.94 ± 0.80	19.67 ± 0.95
CPM44	*C. pilosula* var. *modesta*	PM2		25.56 ± 1.40
CPM45	*C. pilosula* var. *modesta*	PM3	27.35 ± 1.01	

The hybrid ratio was equal to ‘Average ± S.D.’.

**Table 4:** Cultivated *Codonopsis* species in this study.

Voucher No.	Species	Locality	Locality No. *^a^*	Altitude (m)	Date of Collection	Sequence Type (ITS) *^b^*
CP01	*C. pilosula*	Liancai, Longde, Guyuan, Ningxia, China	1	1760	20 July 2016	P1
CP02	*C. pilosula*	Kezhai, Longxi, Dingxi, Gansu, China	2	2220	25 October 2016	P2
CP03	*C. pilosula*	Xiaozhai, Minxian, Dingxi, Gansu, China	3	2550	25 October 2016	P1
CP04	*C. pilosula*	Weixin, Minxian, Dingxi, Gansu, China	4	2225	25 October 2016	P1
CP05	*C. pilosula*	Xijiang, Minxian, Dingxi, Gansu, China	5	2254	25 October 2016	P1
CP06	*C. pilosula*	Meichuan, Minxian, Dingxi, Gansu, China	6	2328	25 October 2016	P1
CP07	*C. pilosula*	Minyang, Minxian, Dingxi, Gansu, China	7	2305	25 October 2016	P1
CP27	*C. pilosula*	Minyang, Minxian, Dingxi, Gansu, China		2305	14 October 2017	P1
CP08	*C. pilosula*	Chabu, Minxian, Dingxi, Gansu, China	8	2313	25 October 2016	P1
CP28	*C. pilosula*	Mazichuan, Minxian, Dingxi, Gansu, China	9	2510	14 October 2017	P1
CP09	*C. pilosula*	Zhongzhai, Minxian, Dingxi, Gansu, China	10	2381	25 October 2016	P1
CP10	*C. pilosula*	Hadapu, Tanchang, Longnan, Gansu, China	11	2281	28 October 2016	P3
CP11	*C. pilosula*	Hadapu, Tanchang, Longnan, Gansu, China		2250	28 October 2016	P1
CP12	*C. pilosula*	Hadapu, Tanchang, Longnan, Gansu, China		2238	28 October 2016	P1
CP13	*C. pilosula*	Hadapu, Tanchang, Longnan, Gansu, China		2233	28 October 2016	P1
CP14	*C. pilosula*	Hadapu, Tanchang, Longnan, Gansu, China		2271	28 October 2016	P1
CP34	*C. pilosula*	Hadapu, Tanchang, Longnan, Gansu, China		2445	14 October 2017	P4
CP35	*C. pilosula*	Hadapu, Tanchang, Longnan, Gansu, China		2435	14 October 2017	P1
CP36	*C. pilosula*	Hadapu, Tanchang, Longnan, Gansu, China		2188	14 October 2017	P1
CP37	*C. pilosula*	Hadapu, Tanchang, Longnan, Gansu, China		2242	14 October 2017	P3
CP15	*C. pilosula*	Awu, Tanchang, Longnan, Gansu, China	12	2421	28 October 2016	P1
CP32	*C. pilosula*	Awu, Tanchang, Longnan, Gansu, China		2351	14 October 2017	P1
CP33	*C. pilosula*	Awu, Tanchang, Longnan, Gansu, China		2329	14 October 2017	P1
CP16	*C. pilosula*	Pangjia, Tanchang, Longnan, Gansu, China	13	2503	28 October 2016	P1
CP17	*C. pilosula*	Pangjia, Tanchang, Longnan, Gansu, China		2368	28 October 2016	P1
CP18	*C. pilosula*	Pangjia, Tanchang, Longnan, Gansu, China		2390	28 October 2016	P1
CP30	*C. pilosula*	Pangjia, Tanchang, Longnan, Gansu, China		2431	14 October 2017	P1
CP31	*C. pilosula*	Pangjia, Tanchang, Longnan, Gansu, China		2456	14 October 2017	P1
CP38	*C. pilosula*	Pangjia, Tanchang, Longnan, Gansu, China		2320	14 October 2017	P5
CP39	*C. pilosula*	Pangjia, Tanchang, Longnan, Gansu, China		2306	14 October 2017	P1
CP19	*C. pilosula*	Lichuan, Tanchang, Longnan, Gansu, China	14	2255	28 October 2016	P1
CP29	*C. pilosula*	Lichuan, Tanchang, Longnan, Gansu, China		2255	14 October 2017	P1
CP20	*C. pilosula*	Lichuan, Tanchang, Longnan, Gansu, China		2286	28 October 2016	P1
CP21	*C. pilosula*	Lichuan, Tanchang, Longnan, Gansu, China		2388	28 October 2016	P1
CP22	*C. pilosula*	Lichuan, Tanchang, Longnan, Gansu, China		2475	28 October 2016	P1
CP23	*C. pilosula*	Lichuan, Tanchang, Longnan, Gansu, China		2314	28 October 2016	P1
CP24	*C. pilosula*	Lichuan, Tanchang, Longnan, Gansu, China		2320	28 October 2016	P1
CP25	*C. pilosula*	Guanzhuang, Yaozhou, Tongchuan, Shaanxi, China	15	880	15 November 2016	P1
CP26	*C. pilosula*	Hongtiguan, Pingshun, Changzhi, Shanxi, China	16	1245	12 March 2017	P4
CPM40	*C. pilosula* var. *modesta*	Danbao, Wenxian, Longnan, Gansu, China	17	895	15 October 2016	PM1
CPM41	*C. pilosula* var. *modesta*	Koutouba, Wenxian, Longnan, Gansu, China	18	1266	15 October 2016	PM1
CPM42	*C. pilosula* var. *modesta*	Shifang, Wenxian, Longnan, Gansu, China	19	995	28 October 2016	PM2
CPM43	*C. pilosula* var. *modesta*	Baoziba, Wenxian, Longnan, Gansu, China	20	1634	28 October 2016	PM2
CPM44	*C. pilosula* var. *modesta*	Baoziba, Wenxian, Longnan, Gansu, China		1480	28 October 2016	PM3
CPM45	*C. pilosula* var. *modesta*	Zhongzhai, Wenxian, Longnan, Gansu, China	21	1361	28 October 2016	PM2
CT46	*C. tangshen*	Banqiao, Enshi, Enshi, Hubei, China	22	1775	18 August 2016	T1

*^a^* Localities of collection are shown in Figure 3; *^b^* the sequence type is indicated in Table 2.

## Supplementary Materials

The following are available online at http://www.mdpi.com/1420-3049/23/7/1565/s1, Figure S1: The thumbnail picture of ITS sequences of 46 *Codonopsis* specimens.
