# Supplementary material for: Study on Hybrid Characteristics of Medicinally Used Cultivated Codonopsis Species Using Ribosomal Internal Transcribed Spacer (ITS) Sequencing
Source: Molecules. 2018 Jun 28;23(7):1565. doi: 10.3390/molecules23071565 (PMC6099391; doi:10.3390/molecules23071565)
Supplement: Supplementary file 1 [file molecules-23-01565-s001.pdf]

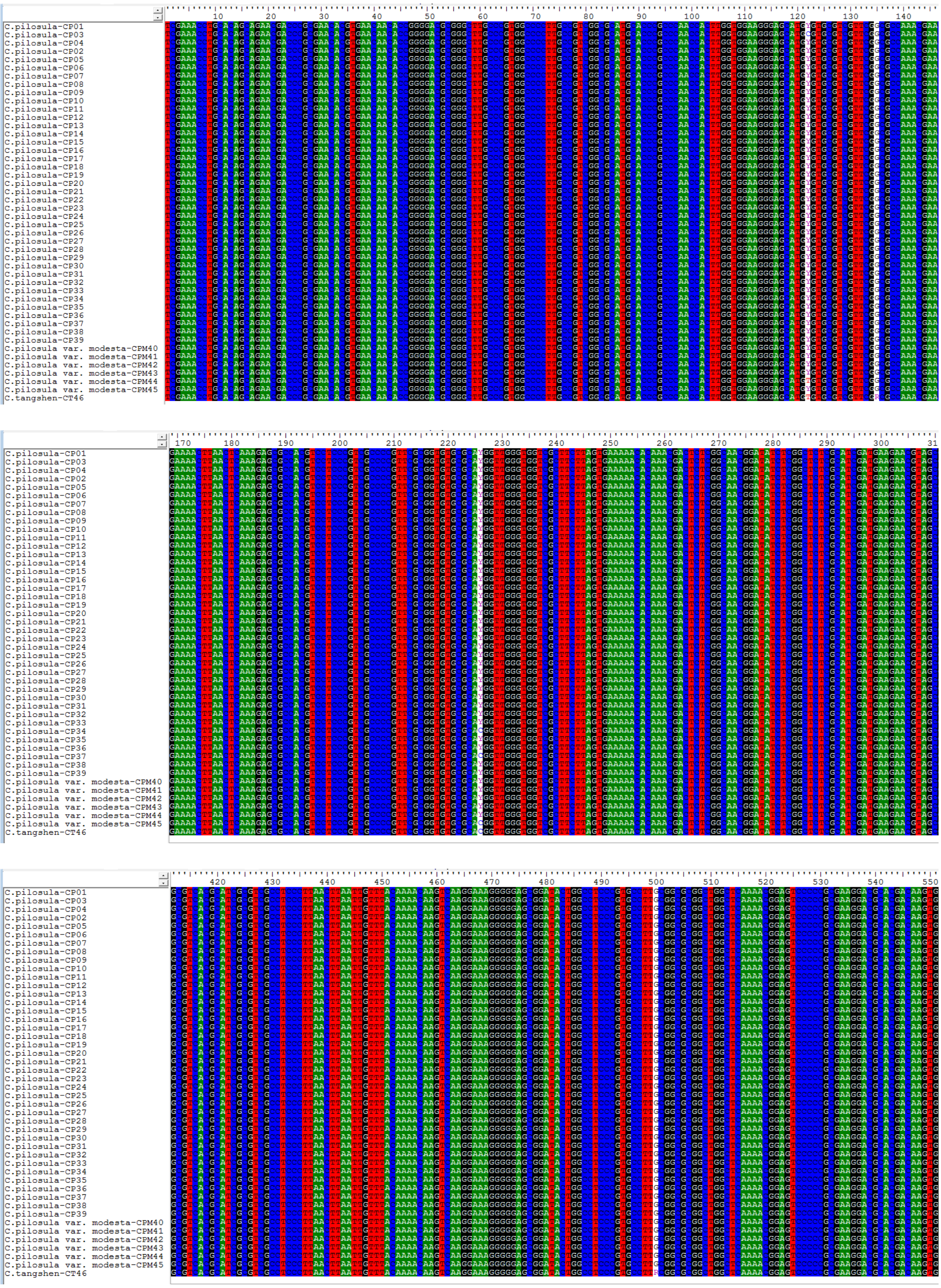

Fig. S1.The thumbnail picture of ITS sequences of 46 *Codonopsis* taxa specimens
The variable base sites were located at 122nd, 135th, 226th and 500th nucleotide positions respectively.
